# Supplementary material for: Maternal Supplementation with N-Acetylcysteine Modulates the Microbiota-Gut-Brain Axis in Offspring of the Poly I:C Rat Model of Schizophrenia
Source: Antioxidants (Basel). 2023 Apr 20;12(4):970. doi: 10.3390/antiox12040970 (PMC10136134; doi:10.3390/antiox12040970)
Supplement: Supplementary file 1 [file antioxidants-12-00970-s001.zip › antioxidants-2225540-supplementary.pdf]

Supplementary Figure S1. Nested bar plot showing gut microbiota composition at the family level in all study groups. Individual gut microbiota composition data for each animal in each study group at the family level. The compositions at family level was relatively homogeneous between animals in each group.

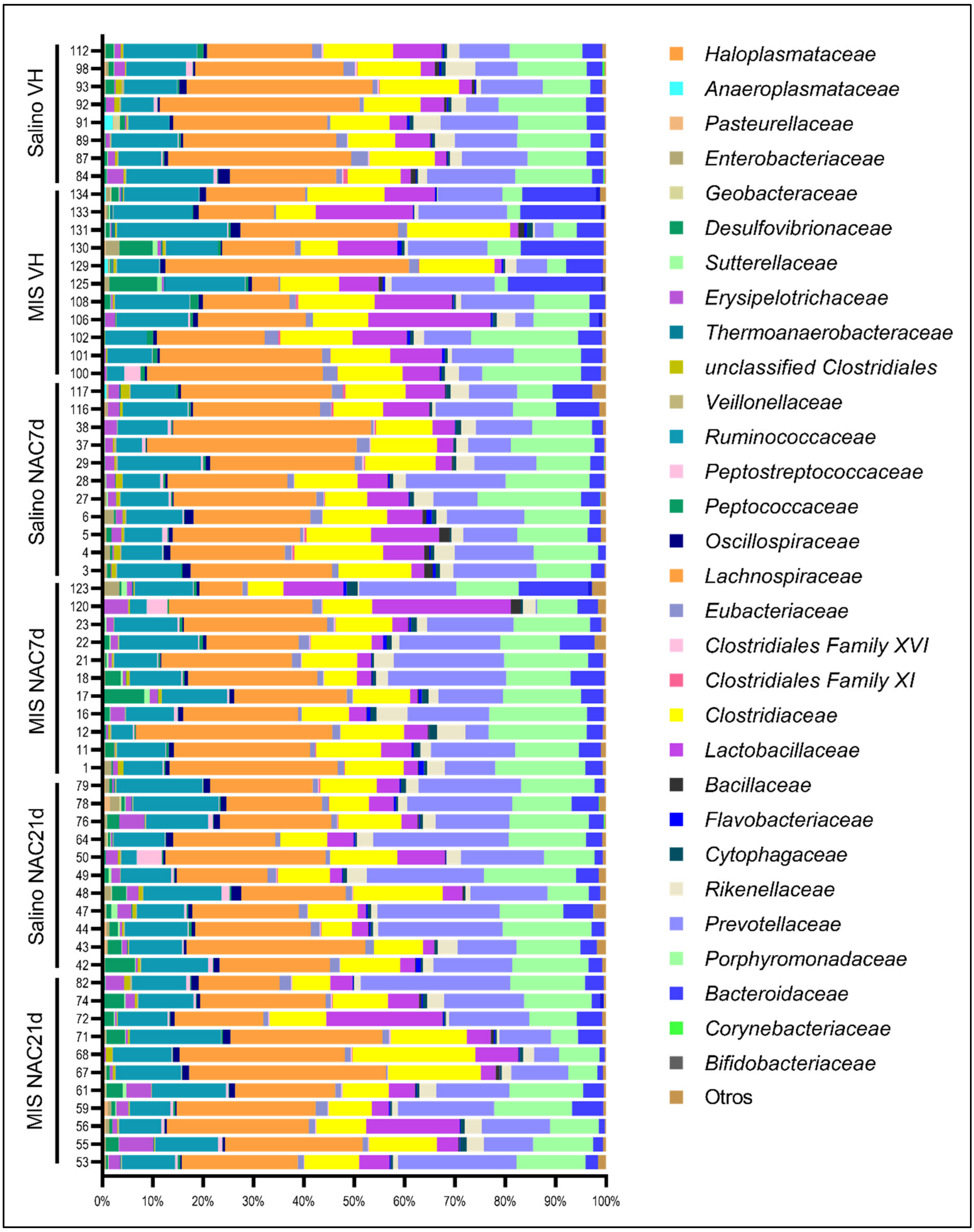

Supplementary Table S1. Data from phyla populations. Table shows the mean ± SEM for each group and the p value as result of the Kruskal-Wallis (KW) analysis (*p < 0.05). η2: size effect. *Firmicutes* showed an interesting interaction with a reduction of bacterias in saline-offspring treated with NAC21 while the opposite pattern was found in MIS-treated animals. An effect of NAC on *Deferribacteres* was found with an increase of bacteria in both groups treated with NAC for 21 days.

Phylum	Sal VH		MIS VH		Sal NAC7d		MIS NAC7d		Sal NAC21d		MIS NAC21d		KW (p-value)	η2
	Mean	SEM	Mean	SEM	Mean	SEM	Mean	SEM	Mean	SEM	Mean	SEM		
Actinobacteria	0.112	0.049	0.126	0.055	0.072	0.028	0.099	0.072	0.086	0.034	0.036	0.018	0.744	0.044
0.Bacteroidetes	32.980	1.505	31.640	2.700	33.560	1.287	39.310	2.841	40.030	2.318	32.530	3.106	**0.052**	**0.175**
Firmicutes	64.870	1.673	64.880	3.574	64.600	1.307	57.440	3.174	56.110	2.258	64.670	3.153	**0.031***	**0.198**
Proteobacteria	1.623	0.288	2.933	1.273	1.383	0.247	2.664	0.792	3.510	0.515	2.557	0.480	0.057	0.171
Cyanobacteria	0.050	0.003	0.000	0.000	0.002	0.002	0.003	0.003	0.018	0.010	0.007	0.004	0.343	0.064
Deferribacteres	0.000	0.000	0.000	0.000	0.000	0.000	0.000	0.000	0.040	0.030	0.085	0.047	**0.033***	0.196
Tenericutes	0.337	0.266	0.378	0.132	0.357	0.228	0.464	0.214	0.133	0.046	0.112	0.094	0.092	0.163
Unclassified Bacteria	0.069	0.055	0.004	0.004	0.006	0.006	0.006	0.006	0.044	0.039	0.000	0.000	0.791	0.016
Verrumicrobia	0.000	0.000	0.027	0.018	0.010	0.008	0.005	0.004	0.010	0.005	0.012	0.009	0.708	0.040

Supplementary Table S2. Data from family populations. Table shows the mean ± SEM for each group and the p value as result of the Kruskal-Wallis analysis (*p < 0.05). η2: size effect.

Family	Sal VH		MIS VH		Sal NAC7d		MIS NAC7d		Sal NAC21d		MIS NAC21d		KW (p value)	η2
	Mean	SEM	Mean	SEM	Mean	SEM	Mean	SEM	Mean	SEM	Mean	SEM		
Haloplasmataceae	0.07	0.06	0.00	0.00	0.01	0.01	0.01	0.01	0.04	0.04	<0.001	<0.001	0.79	0.016
Anaeroplasmataceae	0.27	0.27	0.28	0.11	0.13	0.08	0.07	0.02	0.08	0.03	0.01	0.01	0.06	0.147
Pasteurellaceae	0.19	0.07	0.09	0.04	0.18	0.04	0.14	0.03	0.38	0.09	0.29	0.10	0.033**	0.226
Enterobacteriaceae	0.19	0.07	0.51	0.24	0.59	0.19	0.50	0.28	0.64	0.17	0.35	0.06	0.12	0.132
Geobacteraceae	0.19	0.15	0.06	0.03	0.02	0.01	0.04	0.04	0.11	0.04	0.07	0.02	0.36	0.083
Desulfovibrionaceae	0.90	0.20	1.92	0.94	0.29	0.10	1.55	0.71	1.76	0.51	1.68	0.44	0.018*	0.218
Sutterellaceae	0.08	0.03	0.25	0.11	0.05	0.02	0.32	0.12	0.23	0.11	0.14	0.06	0.43	0.090
Veillonellaceae	0.24	0.05	0.08	0.02	0.22	0.03	0.12	0.02	0.16	0.02	0.15	0.03	0.004**	0.304
Erysipelotrichaceae	1.36	0.36	0.43	0.16	1.57	0.23	1.45	0.41	1.63	0.43	2.24	0.64	0.014*	0.216
Thermoanaero bacteraceae	0.01	0.01	0.25	0.07	0.06	0.03	0.06	0.03	0.08	0.03	0.04	0.02	0.08	0.159
Unclassified *Clostridiales*	0.46	0.14	0.21	0.06	0.70	0.16	0.36	0.10	0.30	0.09	0.39	0.13	0.14	0.120
Ruminococcaceae	11.32	1.28	12.43	1.53	9.98	0.96	9.62	1.09	11.90	1.20	11.69	0.87	0.44	0.089
Peptostreptococcaceae	0.44	0.16	0.38	0.29	0.43	0.10	0.67	0.35	0.94	0.43	0.46	0.07	0.05	0.194
Peptococcaceae	0.33	0.13	0.66	0.15	0.29	0.04	0.37	0.06	0.24	0.04	0.21	0.04	0.07	0.215
Oscillospiraceae	0.90	0.23	0.90	0.14	0.76	0.18	0.57	0.11	1.00	0.15	0.89	0.15	0.41	0.079
Lachnospiraceae	30.66	2.46	23.74	3.61	28.74	1.89	25.44	2.38	23.08	1.65	26.05	2.05	0.12	0.179
Eubacteriaceae	1.64	0.32	1.43	0.26	1.57	0.21	1.34	0.12	1.39	0.11	1.28	0.18	0.86	0.029
Clostridiales Family XVI	0.24	0.05	0.00	0.00	0.16	0.03	0.12	0.03	0.09	0.02	0.23	0.03	<0.001***	0.378
Clostridiales Family XI	0.15	0.10	0.12	0.06	0.21	0.06	0.08	0.03	0.10	0.03	0.04	0.02	0.60	0.058
Clostridiaceae	12.25	0.71	12.98	1.11	12.60	0.73	10.49	0.64	10.90	0.93	12.67	1.51	0.27	0.112
Lactobacillaceae	4.26	0.95	10.90	2.06	6.67	0.99	6.26	2.29	3.97	0.65	7.92	2.00	0.029*	0.243
Bacillaceae	0.44	0.13	0.22	0.12	0.57	0.21	0.28	0.18	0.11	0.02	0.24	0.08	0.12	0.127
Flavobacteriaceae	0.32	0.04	0.43	0.06	0.24	0.10	0.45	0.10	0.29	0.09	0.18	0.02	0.026*	0.208
Cytophagaceae	0.51	0.09	0.51	0.11	0.73	0.07	1.09	0.16	0.51	0.08	0.58	0.10	0.014*	0.229
Rikenellaceae	3.22	0.61	1.45	0.33	2.78	0.29	2.92	0.53	2.37	0.32	2.03	0.36	0.06	0.157
Prevotellaceae	11.90	1.27	10.99	1.78	13.07	1.11	14.76	2.19	19.48	1.50	15.31	2.07	0.022*	0.217
Porphyromonadaceae	13.76	0.84	9.11	2.02	12.97	1.17	14.70	1.08	13.83	0.93	11.11	1.10	0.09	0.135
Bacteroidaceae	3.09	0.22	8.99	1.93	3.56	0.72	5.13	1.04	3.30	0.42	3.12	0.53	0.009**	0.251
Corynebacteriaceae	0.10	0.05	0.00	0.00	0.03	0.02	0.00	0.00	0.04	0.03	0.00	0.00	0.006**	0.298
Bifidobacteriaceae	0.00	0.00	0.07	0.05	0.00	0.00	0.00	0.00	0.00	0.00	0.00	0.00	0.004**	0.243

Supplementary Table S3. Genus and species proportions. This table shows the percentage (%) of different members of genus and species in each group. η2: size effect. Data are shown as mean ± SEM. In the KW column, results from the Kruskal-Wallis analysis are shown [*p < 0.05. **p < 0.01. ***p < 0.001].

Genus/Specie	Sal VH		MIS VH		Sal NAC7d		MIS NAC7d		Sal NAC21d		MIS NAC21d		KW (p value)	η2
	Mean	SEM	Mean	SEM	Mean	SEM	Mean	SEM	Mean	SEM	Mean	SEM		
Bifidobacterium animalis	0.00	0.00	0.05	0.05	0.00	0.00	0.00	0.00	0.00	0.00	0.00	0.00	0.09	0.243
Corynebacterium stationis	0.10	0.05	0.00	0.00	0.03	0.01	0.00	0.00	0.05	0.03	0.00	0.00	**0.009****	**0.298**
***Bacteroides* (gen.)**	0.59	0.18	5.39	1.94	1.35	0.43	2.02	0.71	0.85	0.28	1.25	0.41	0.67	0.042
Bacteroides acidifaciens	0.00	0.00	0.09	0.04	0.03	0.02	0.00	0.00	0.01	0.01	0.00	0.00	**0.038***	**0.280**
Bacteroides dorei	0.59	0.18	3.26	1.48	0.97	0.31	1.40	0.40	0.84	0.28	1.13	0.41	0.93	0.027
Bacteroides vulgatus	0.00	0.00	0.26	0.15	0.14	0.07	0.32	0.30	0.00	0.00	0.08	0.04	0.07	0.207
Bacteroides uniformis	0.00	0.00	1.50	0.76	0.08	0.06	0.18	0.13	0.00	0.00	0.03	0.01	**0.014***	**0.263**
Parabacteroides distasonis	0.01	0.01	0.12	0.07	0.06	0.02	0.02	0.01	0.00	0.00	0.00	0.00	**0.026***	**0.205**
***Prevotella* (gen.)**	2.61	0.63	2.45	0.54	2.66	0.53	3.52	0.84	4.47	0.73	3.03	0.48	0.25	0.115
***Alistipes* (gen.)**	1.14	0.42	0.83	0.23	0.69	0.29	0.63	0.24	1.00	0.25	0.38	0.16	0.19	0.124
***Lactobacillus* (gen.)**	4.00	0.94	10.79	2.05	6.61	1.01	6.10	2.27	3.59	0.64	7.70	2.00	**0.017***	**0.261**
Lactobacillus hominis	0.03	0.03	0.05	0.03	0.19	0.09	0.09	0.03	0.15	0.10	0.04	0.02	0.20	0.112
Lactobacillus intestinalis	0.01	0.01	0.14	0.04	0.56	0.14	0.62	0.25	0.24	0.08	0.27	0.11	**0.003****	**0.350**
Lactobacillus animalis	0.01	0.01	0.14	0.04	0.09	0.04	0.08	0.04	0.01	0.01	0.22	0.09	**0.023***	**0.207**
Lactobacillus johnsonii	0.81	0.21	1.24	0.36	0.51	0.12	0.42	0.24	0.48	0.09	0.51	0.10	0.07	0.199
Lactobacillus kitasatonis	0.00	0.00	0.13	0.06	0.00	0.00	0.01	0.01	0.00	0.00	0.00	0.00	**0.007****	**0.174**
Lactobacillus murinus	0.35	0.13	1.08	0.28	0.81	0.22	0.75	0.25	0.37	0.10	1.13	0.46	0.22	0.128
Lactobacillus reuteri	0.11	0.07	0.26	0.09	0.18	0.03	0.15	0.09	0.10	0.04	0.14	0.02	0.06	0.196
Lactobacillus vaginalis	0.31	0.11	0.56	0.24	0.55	0.08	0.51	0.32	0.31	0.07	0.37	0.06	0.10	0.155
Candidatus arthromitus	0.04	0.04	0.01	0.01	0.09	0.04	0.04	0.02	0.15	0.10	0.08	0.05	0.19	0.178
***Clostridium* (gen.)**	7.50	0.56	7.91	0.76	7.40	0.61	6.35	0.37	6.81	0.88	8.24	1.07	0.42	0.084
Clostridium hiranonis	0.05	0.03	0.02	0.01	0.05	0.02	0.07	0.03	0.11	0.05	0.08	0.03	0.30	0.203
Clostridium sp.	2.39	0.25	2.23	0.36	2.36	0.26	1.84	0.17	1.68	0.27	2.77	0.51	0.16	0.120
Clostridium vincentii	0.05	0.02	0.03	0.03	0.02	0.01	0.15	0.04	0.33	0.11	0.14	0.04	**<0.001*****	**0.326**
***Eubacterium* (gen.)**	0.10	0.04	0.10	0.05	0.28	0.12	0.15	0.10	0.15	0.03	0.10	0.03	0.63	0.073
***Acetatifactor* (gen.)**	0.14	0.03	0.03	0.01	0.12	0.03	0.17	0.04	0.18	0.05	0.15	0.04	**0.043***	**0.182**
***Coprococcus* (gen.)**	1.01	0.44	0.35	0.15	0.66	0.35	0.59	0.26	0.40	0.13	0.28	0.16	0.61	0.044
Eubacterium hadrum	0.34	0.30	0.02	0.01	0.01	0.01	0.09	0.05	0.26	0.13	0.02	0.01	0.14	0.180
***Lachnoanaerobaculum* (gen.)**	0.44	0.17	0.05	0.03	0.35	0.09	0.44	0.10	0.24	0.07	0.28	0.10	**0.030***	**0.232**
***Lachnoclostridium* (gen.)**	0.25	0.09	0.19	0.07	0.20	0.07	0.19	0.06	0.09	0.04	0.21	0.06	0.66	0.022
***Roseburia* (gen.)**	0.31	0.16	0.05	0.05	0.23	0.07	0.26	0.12	0.38	0.13	0.83	0.29	**0.001****	**0.372**
***Ruminococcus* (gen.)**	4.61	0.78	4.99	0.89	5.44	0.56	4.31	0.70	3.41	0.47	4.23	0.64	0.25	0.176
***Oscillibacter* (gen.)**	0.11	0.05	0.12	0.03	0.05	0.02	0.05	0.02	0.13	0.02	0.11	0.03	0.11	0.153

***Faecalibacterium* (gen.)**	5.19	0.93	7.30	1.10	3.87	0.47	4.23	0.63	5.47	0.60	5.98	0.72	0.05	0.201
***Ruminiclostridium* (gen.)**	0.09	0.01	0.04	0.02	0.07	0.01	0.06	0.02	0.07	0.02	0.05	0.02	0.24	0.137
***Turicibacter* (gen.)**	0.55	0.20	0.18	0.11	0.65	0.12	0.58	0.20	0.60	0.23	1.27	0.46	**0.023***	**0.228**
Parasutterella excrementihominis	0.09	0.03	0.25	0.11	0.05	0.02	0.31	0.12	0.23	0.11	0.15	0.06	0.52	0.090
Bilophila Wadsworthia	0.73	0.20	1.89	0.95	0.05	0.02	0.77	0.43	0.38	0.16	0.70	0.38	**0.009****	**0.241**
***Desulfovibrio* (gen.)**	0.11	0.05	0.00	0.00	0.15	0.08	0.01	0.01	0.45	0.22	0.15	0.05	**0.014***	**0.259**
